# Patient Satisfaction, Functional Outcomes, and Implant Survivorship in Patients Undergoing Customized Unicompartmental Knee Arthroplasty

**DOI:** 10.3390/jpm11080753

**Published:** 2021-07-30

**Authors:** Cyrus Anthony Pumilia, Lennart Schroeder, Nana O. Sarpong, Gregory Martin

**Affiliations:** 1 College of Medicine, University of Central Florida, Orlando, FL 32816, USA; 2 Department of General, Trauma and Reconstructive Surgery, University Hospital, Ludwig Maximilians University, 81377 Munich, Germany; lennartschroeder@gmx.de; 3 Columbia University Medical Center, Department of Orthopedic Surgery, New York—Presbyterian Hospital, Columbia University, New York, NY 10032, USA; nosarp1@gmail.com; 4 Department of Orthopedic Surgery, Personalized Orthopaedics of the Palm Beaches, Boynton Beach, FL 33437, USA; gm277@yahoo.com

**Keywords:** patient-specific, individualized, 3D-printing, unicondylar knee arthroplasty, unicompartmental knee replacement, unicondylar knee replacement, partial knee arthroplasty, partial knee replacement, UKA, UKR

## Abstract

Customized unicompartmental knee arthroplasty (C-UKA) utilizes implants manufactured on an individual patient basis, derived from pre-operative computed tomography images in an effort to more closely approximate the natural anatomy of the knee. The outcomes from 349 medial and lateral fixed-bearing C-UKA were reviewed. Implant survivorship analysis was conducted via retrospective chart review, and follow-up analysis was conducted via a single postoperative phone call or email. The rate of follow-up was 69% (242 knees). The average age at surgery was 71.1 years and the average body mass index was 28.8 kg/m^2^. Seven revision arthroplasties (2.1%) had knowingly been performed at an average of 1.9 years postoperatively (range: 0.1–3.9 years), resulting in an implant survivorship of 97.9% at an average follow-up of 4.2 years (range: 0.1–8.7) and 97.9% at an average of 4.8 years (range: 2.0–8.7) when knees with less than two years of follow-up were excluded. The reasons for revision were implant loosening (one knee), infection (two knees), progression of osteoarthritis (two knees), and unknown reasons (two knees). The average KOOS, JR. interval score was 84 (SD: 14.4). Of those able to be contacted for follow-up analysis, 67% were “very satisfied,” 26% were “satisfied,” 4% were “neutral,” 2% were “dissatisfied,” and 1% were “very dissatisfied.” When asked if the knee felt “natural,” 60% responded with “always,” 35% responded with “sometimes,” and 5% responded with “never.” After analyzing a large cohort of C-UKA, we found favorable rates of survivorship, satisfaction, and patient-reported functional outcomes.

## 1. Introduction

Unicompartmental knee arthroplasty (UKA) was first pioneered in the 1940s and 1950s by Campbell, McKeever, and MacIntosh using interpositional tibial plateau prostheses [1,2,3]. Their original reports demonstrated improvements in pain and function through prosthetic replacement of degenerated joint compartments and correction of varus or valgus deformities. Presently, UKA serves as a viable surgical alternative to total knee arthroplasty (TKA) when joint degeneration is limited to either the medial or lateral tibiofemoral compartment. Though UKA has undergone periods of criticism since its inception, namely, questioning its survival in comparison to TKA [4,5], it may offer faster recovery [6,7,8], reduced complication rates [7,8,9,10], improved patient-reported functional outcomes [11,12,13], and a more normal feeling knee [14,15] in appropriately selected patients. The importance of continuing to study UKA and its technological developments is highlighted by the significant and increasing healthcare burden that osteoarthritis (OA) poses across the world and the increasing number of patients with OA-related knee disorders who seek to maintain a high level of activity [16,17,18,19].

One of the more recent technological developments in arthroplasty has been the introduction of customized, or patient-specific, implants. In contrast to the traditional method of selecting implant size and geometry from an available set of options, these implants are manufactured on an individual basis from a three-dimensional rendering of pre-operative computed tomography (CT) imaging. Their development originated from the high variability seen in distal femoral and proximal tibial bone geometry [16,17,18,19], as well as the increasing focus on restoring the natural knee anatomy with arthroplasty in recent years [20,21]. In theory, a closer approximation of the natural anatomy would provide for improved kinematics, as shown in customized TKA (C-TKA) [22,23]. Since first appearing in the literature in 2009 [24], C-UKA has shown some potential improvements over conventional UKA, though kinematic studies have not been conducted. Namely, C-UKA has shown improved fit of the tibial component [25,26] and reduced contact stress on the opposite tibiofemoral compartment [27].

To date, there are only limited data on the clinical outcomes of C-UKA. Previous studies have shown satisfactory radiographic outcomes [28], as well as satisfactory short-term clinical results [26,29]. Only one study, to the best of the authors’ knowledge, has investigated the outcomes of C-UKA at the mid-term follow-up [30]. The aim of the present study was to retrospectively analyze patient satisfaction, PROMs, and implant survivorship in a large patient cohort with C-UKA at the mid-term follow-up.

## 2. Materials and Methods

After obtaining approval from the institutional review board, all patients who had undergone fixed-bearing C-UKA (iUni, ConforMIS, Billerica, MA, USA) by a single surgeon between March 2010 and August 2017 were identified. Surgery was performed using customized, or patient-specific, cutting guides provided by the manufacturer. Either a medial or lateral parapatellar approach was utilized. Patient selection for UKA began with four-view plain radiographs of the knee (weightbearing anteroposterior, weightbearing lateral, Rosenberg, and Sunrise views). If joint degeneration appeared to be contained to solely the medial or lateral tibiofemoral compartment, the patient was considered for UKA and further evaluated with a computed topography arthrogram (CT-arthrogram). If the CT-arthrogram confirmed unicompartmental disease and the patient met the indications, UKA was offered. The indications in our patient cohort included an intact anterior cruciate ligament, a body mass index (BMI) below 40, non-inflammatory arthritis, a correctable varus deformity of less than 10 degrees or a correctable valgus deformity of less than 5 degrees, a flexion contracture less than 15 degrees, and a range of motion greater than 90 degrees, some of which were described by Scott et al. [31,32,33,34]. No age minimum was utilized. There were no significant changes to the selection or surgical protocols during the time period of the study. Approximately 20–25% of the surgeon’s yearly knee arthroplasty collective consisted of UKA.

Patient demographics, surgical variables, and intra- and postoperative complications, as well as re-operations, were recorded from electronic medical records. To assess patient satisfaction, functional outcomes, and implant survivorship, a single postoperative follow-up questionnaire was administered by phone. Patients who were unable to be contacted by phone were contacted by email, through which questionnaires were administered. If contact could not be established after three attempts, the patient was classified as non-contactable. 

The KOOS, JR. [35] questionnaire was administered during follow-up to evaluate PROMs. This seven-item PROM combines questions on pain, symptoms, and functional limitations to provide a single score ranging from 0 to 100, with higher scores representing a healthier knee. To assess patient satisfaction, the study subjects were asked to respond to the question “Are you satisfied with your knee replacement?” on a five-item word rating scale of very satisfied, satisfied, neutral, dissatisfied, and very dissatisfied. To survey patient-perceived feelings of the C-UKA, the study participants were asked if their replaced knee felt “natural,” with answer choices including “always,” “sometimes,” or “never.” The average time of follow-up was determined after all patients were contacted by phone or classified as non-contactable.

Two separate patient cohort analyses were performed: A follow-up analysis and an implant survivorship analysis. Patients who had died were excluded from both analyses. Follow-up analysis consisted of questionnaire data obtained from contactable patients. In the contactable patients, implant survivorship analysis was performed by asking if revision had been performed during the follow-up phone call. The time point at which the phone call was conducted was considered the follow-up length. In non-contactable patients, implant survivorship analysis was performed by chart abstraction to identify if revision surgery had been recorded in the EMR. The last documented clinic visit without recorded revision surgery, as confirmed by patient history, examination, and imaging, was considered the follow-up length. Implant survivorship analysis was divided into two groups based upon follow-up length. One group consisted of all implanted knees and the other consisted of only knees with greater than or equal to two years of follow-up. Component revision for any reason in both contactable and non-contactable patients was defined as the implant survival endpoint. Patients who underwent revision, did not consent to participation, were non-contactable, or were confirmed as deceased were excluded from the follow-up analysis.

To examine the significance of contingencies, Fisher’s exact test was performed and Student’s *t*-test was used to determine nonrandom associations between the analyzed variables.

## 3. Results

**Figure 1:**
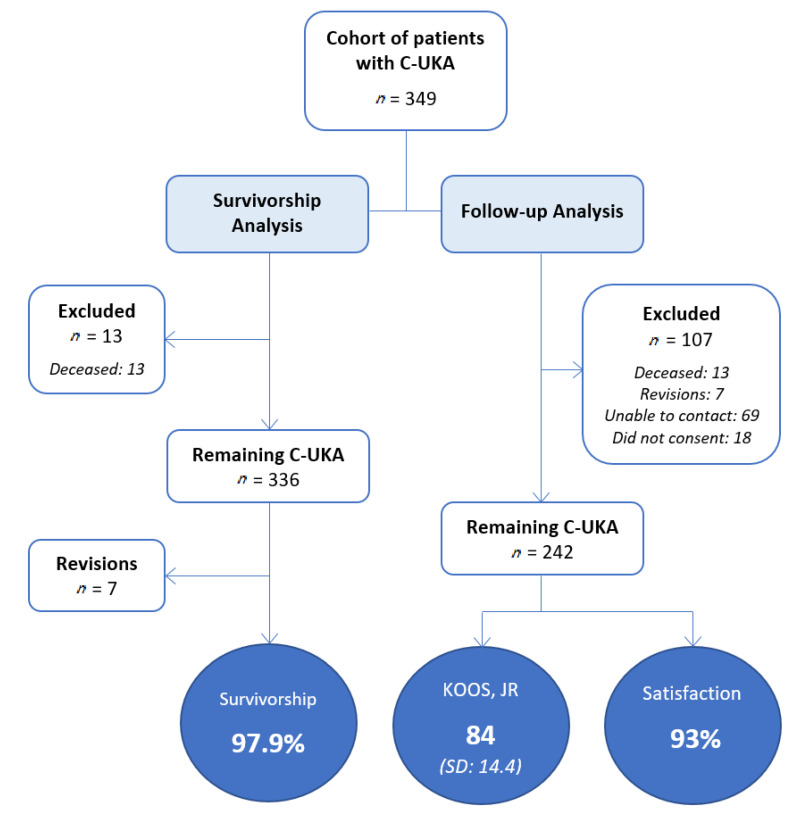
Flowchart of survivorship and follow-up analyses.

The study population consisted of 297 patients (349 knees), of which 118 (40%) were female. The average age at surgery was 71.1 years (SD: 9.2 years) with a mean BMI of 28.8 kg/m^2^ (SD: 4.7) (Table 1). Of the total C-UKA, 287 (82%) were implanted medially and 62 (18%) laterally. At the time of follow-up, 12 patients (13 knees) (3.7%) had died and were therefore excluded from the survivorship and follow-up analyses. One patient died shortly after the UKA procedure, presumably from cardiopulmonary arrest. Death notice for the remaining 11 patients was received during attempted phone contact with no further investigation conducted. At the time of follow-up, seven revision arthroplasties (2.1%) had knowingly been performed at an average of 1.9 years postoperatively (range of 0.1–3.9 years). The reasons for revision were implant loosening (one knee), infection (two knees), progression of osteoarthritis leading to the implantation of a total knee replacement (two knees), and unknown reasons (two knees). This resulted in an implant survivorship of 97.9% at the time of phone follow-up or last documented clinic visit in all knees (Figure 1). When all knees with less than two years of follow-up were excluded from the implant survivorship analysis, 304 knees (87.1%) were left with an average follow-up length of 4.8 years (range of 2.0–8.7 years). Thirteen of these knees (3.7%) were known to be deceased. This left 291 knees (83.4%) remaining, upon which six revisions were reported (2.1%), also resulting in an implant survivorship of 97.9%.

Of the 349 knees enrolled, 242 (69%) were able to be contacted, consented for participation, and were therefore included in the follow-up analysis (Figure 1). Of those not included in the follow-up analysis, 79% (69) were unable to be contacted and 21% (18) did not consent to participation. The average follow-up, as determined by the time from preoperative hospital admission to follow-up contact or last documented clinic visit, was found to be 4.2 years (range of 0.1–8.7 years). Medical records revealed two postoperative complications related to the UKA procedure. One patient developed a hematoma postoperatively and was brought back to the operating room for wound irrigation, debridement, and tibial liner exchange. The other patient was brought back to the operating room for wound irrigation, debridement, and primary closure after a fall causing wound dehiscence at five weeks postoperation. 

**Figure 2:**
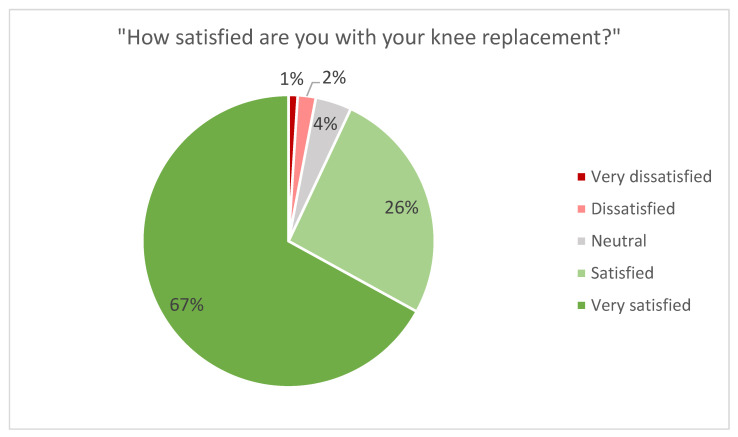
Patient satisfaction with C-UKA.

**Figure 3:**
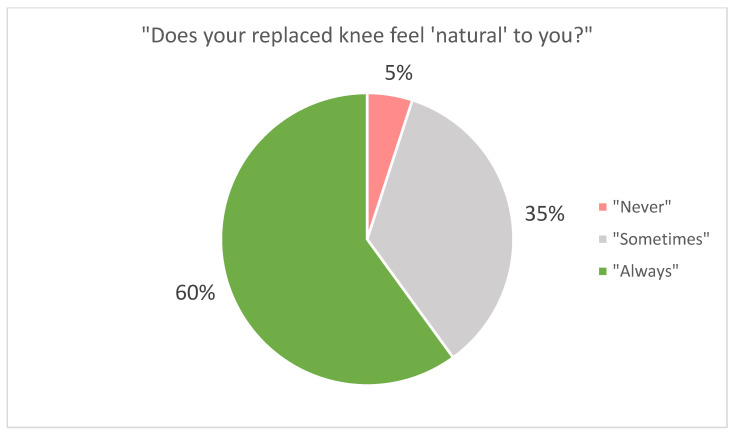
Responses to “Does your replaced knee feel ‘natural’ to you?”

The evaluation of functional outcomes, as measured by the KOOS, JR, showed an average score of 84 (SD: 14.4). When assessing patient satisfaction, 67% of patients were “very satisfied,” 26% were “satisfied,” 4% were “neutral,” 2% were “dissatisfied,” and 1% were “very dissatisfied” (Figure 2). When asked if the knee felt “natural,” 60% of the study participants responded that their knee “always” felt natural, 35% responded that their knee “sometimes” felt natural, and 5% responded that their knee “never” felt natural (Figure 3).

## 4. Discussion

Innovation in prosthesis design and implantation has long been the norm in arthroplasty. In recent years, numerous new UKA technologies, such as customized implantation, have been developed and are becoming increasingly reported in the literature [36]. Though C-UKA has demonstrated favorable characteristics, such as improved component fit [25,26] and reduced opposite compartment contact stress [27], its clinical outcomes have yet to be established at mid- or long-term follow-up. To the best of the authors’ knowledge, this patient cohort is the largest to be studied after C-UKA. We retrospectively analyzed the survival, satisfaction, and PROMs of 349 knees at an average follow-up of 4.2 years. 

Implant survivorship is one of the most common concerns with UKA. Data from the Australian Orthopaedic Association National Joint Replacement Registry show revision rates of 5.2% at three years and 7.5% at five years in fixed-bearing UKA [37], similar to those of the National Joint Registry for England, Wales, and Northern Ireland at 3.43% and 5.36%, respectively [38]. Data from the New Zealand Joint Registry show a revision rate of 4.4% at four years [39]. The data available in the literature for the revision rate of fixed-bearing UKA (combined medial and lateral) include 10% at 5.5 years from Middleton et al. [40], 7.8% at 5.7 years from Biswal et al. [41], and 4% at five years from Whittaker et al. [42]. Though accurate comparison of data is not feasible, especially considering our retrospective study design, as well as the potential variance in the surgeon threshold for revision, an implant survivorship of 97.9% was observed in our cohort of C-UKA at follow-up of 4.2 years. 

Survivorship in C-UKA has only been reported by two previous studies. In 2018, Talmo et al. [30] found a revision rate of 25.2% in a retrospective analysis of 115 medial C-UKAs at follow up of 4.5 years (average time to implant failure of 2.8 years). These findings were not echoed by our study, or by Demange et al. [26], who found a rate of 3% at 3.1 years in a prospective cohort of 33 lateral C-UKAs. The most common reason for revision reported by Talmo et al. [30] was aseptic loosening (75.9%), which was a less common reason for revision in our study (14%). Their data do not suggest a clear reason for this discrepancy. Though the average age in their study was much lower (54 vs. 71 years), Demange et al. [26] mirrored our findings with a similarly low average age of 59 years. The average BMI of all studies was similar, ranging from 28.7 to 29 kg/m^2^. The selection criteria of Talmo et al. [30] were not reported and therefore may have differed. Furthermore, patient activity levels were not reported and may have also contributed to the discrepancy in survival if their cohort was significantly more active than ours or that of Demange et al. [26]. Comparison between studies is further limited in that both consist of single-surgeon C-UKA data. The reported technique did not differ substantially between surgeons, and the data of Talmo et al. [30] suggest that surgeon experience did not contribute (as evidenced by substantial surgeon experience in UKA and no clear downward trend in the failure rate as experience with C-UKA increased). Nevertheless, there is a possibility that minor differences in utilization of the customized implantation contributed to the discrepancy in the results. 

To the best of the authors’ knowledge, the satisfaction rates in C-UKA have not previously been reported in the literature. Satisfaction rates have been reported in C-TKA, though with Reimann et al. [43] showing a significant increase in comparison to conventional TKA. Previous studies investigating conventional, fixed-bearing UKA have reported similar satisfaction rates to those of the present study. Biswal et al. [41] reported a satisfaction rate of 92% in a cohort of 128 medial and lateral UKAs at follow-up of 5.7 years. Middleton et al. [40] reported the same satisfaction rate of 92% in a cohort of 129 medial and lateral UKAs at follow-up of 5.5 years. We report a satisfaction rate of 93% at follow-up of 4.2 years. 

Superior functional outcomes, as assessed by PROMs, have often been cited as an advantage of UKA over TKA [11,12]. Functional outcomes were assessed in our study using the KOOS, JR., a validated PROM in joint replacement [35], resulting in an average interval score of 84 out of 100 (SD: 14.4). Though no previous studies have reported KOOS, JR. scores after UKA, normative data collected for subjects aged 18–64 years with healthy knees show a mean score of 92.3 (SD: 11.7) that decreases with age and female sex to 91.5 (SD: 12.1) in 56–64-year-old males and 86.6 (SD: 14.6) in 56–64-year-old females [44]. Further reference may be provided by converting KOOS, JR. scores to equivalent Oxford Knee Scores (OKS) [45] using the PROM crosswalk created by Polascik et al. [46]. In their study, they provided a conversion table and demonstrated similar sample means and distributions between the true and derived PROM scores. It is important to note that this conversion may be limited in converting sample means, as opposed to individual scores, and that it has only been validated in a single study population. Nonetheless, it may be able to provide context for the results of the present study when one is not familiar with the KOOS, JR. Accordingly, the mean KOOS, JR. score of 84 in our study equates to an OKS of 44 (out of 48). For reference, Middleton et al. [40] reported a mean OKS of 38 in 129 fixed-bearing UKAs at 5.5 years, Pandit et al. [47] reported a mean OKS of 41.3 in 1000 mobile-bearing UKAs at 5.6 years, and the New Zealand Joint Registry reported a mean OKS of 41.65 in a cohort of 3112 mixed mobile- and fixed-bearing UKAs at five years [39]. Direct comparison of C-UKA and conventional UKA in future studies may provide more insight into the effects of C-UKA on functional outcomes. 

Future studies that directly compare C-UKA to conventional UKA may also provide insight into where C-UKA could be able to provide advantages, if any, in the decision making between UKA and TKA. The primary concern in the use of UKA over TKA is implant survivorship. For UKA to be worthwhile in any individual patient, it must provide a large enough margin of benefit over TKA for a long enough period of time, as revision to TKA comes at a cost to the patient and may have slightly inferior outcomes to that of primary TKA [48,49]. With UKA often being selected for improved functional outcomes [11,12,13] and a more normal feeling knee [14,15], the theorized closer anatomic approximation and more natural kinematics in C-UKA may be able to provide said margin of benefit if its theory translates into long-term clinical results. Kinematics have yet to be investigated in C-UKA, though they have been investigated in C-TKA, demonstrating improved femoral rollback and improved femoral internal rotation at full extension (i.e., the “screw-home” mechanism) over conventional TKA [22,23]. A large percentage of patients in our study (95%) reported that their knee “always” or “sometimes” felt “natural,” though without comparison to another patient cohort, conclusions are difficult to draw. However, the direction of the results may indicate a successful restoration of patients’ perceived natural feelings of the knee, which may have been a contributing factor for the high satisfaction rate observed. C-UKA may also have an impact on how long the benefits of UKA can be provided, given its potential effects on two of the most common causes of implant failure in UKA: Progression of osteoarthritis and aseptic loosening [50,51]. Biomechanical analysis of medial C-UKA has shown reduced contact stress on the lateral compartment [27], suggesting possible reductions in progression of osteoarthritis. Anatomic studies in C-UKA have shown significantly greater tibial component coverage of the cortical rim [25,26], which may reduce risk for component loosening via tibial bone resorption [52,53], as the component can rely more on the strength of cortical bone as compared to that of weaker, cancellous bone. Though the survivorship shown in our study was favorable, imaging studies were not included in our analysis, and therefore, the above two causes of implant failure cannot be assessed. Clinical investigation and longer-term follow-up of the potential benefits described above will be needed to draw concrete conclusions. 

Multiple limitations of the present study must be addressed. Without a control group, direct comparison of C-UKA to conventional UKA in our cohort was not possible, thereby limiting conclusions. Furthermore, the inherent shortcomings in the retrospective design of this study may have limited the findings. Though the retrospective design allowed for a larger cohort than would have otherwise been possible, loss to follow-up may have introduced attrition bias, should those subjects have had different outcomes than those analyzed. This effect would likely be more pronounced in the follow-up analysis, as 31% of the subjects were unable to be contacted. The survivorship analysis accounted for 96% of subjects (the remaining being deceased) and was conducted from either phone follow-up or chart documentation, with the follow-up length recorded as either the time of the phone follow-up or the last documented clinic visit. Nevertheless, the possibility exists that non-contactable patients in this analysis who were only analyzed via internal medical records may have sought care elsewhere after their last documented clinic visit. It is unknown whether the loss to follow-up seen in this study was due to subject unwillingness to accept contact or if contact never reached those subjects. The average age in our cohort was 71.1 years, so it may be likely that a significant portion of uncontactable patients were unknowingly deceased or had outdated contact information. Furthermore, the large range of follow-up lengths (0.1–8.7 years) may be seen as a potential limitation to the survivorship analysis. This study was carried out in this fashion so as to avoid any exclusion bias, especially that of missing early revisions, as demonstrated by our average time to revision of 1.9 years.

Additionally, our data were only that of a single surgeon, whose patient selection process, experience in UKA, and surgical volume may have played a large role in the results [34,54,55,56]. Specifically, the surgeon in the present study utilized a CT arthrogram in the selection process, which may not be used at all institutions. The yearly volume was greater than 50 UKAs and previous experience with the studied C-UKA implantation system was high. Though our patient-reported outcomes were good, the threshold for revision to TKA may vary among surgeons and has the potential to have contributed to the observed survivorship rates. Furthermore, the patient population that commonly presents to this center and their level of medical comorbidities, as well as administration of PROMs over the phone, may have influenced outcomes and could limit comparison to other studies. 

## 5. Conclusions

After retrospectively analyzing a large cohort of customized unicompartmental knee arthroplasties, we found favorable rates of survivorship, satisfaction, and patient-reported functional outcomes. Though our cohort showed favorable results, these findings may have been limited by the retrospective study design and do not provide insight into how customized unicompartmental knee arthroplasty may compare to other methods. Future studies may be able to provide longer follow-up times, a broader range of patient populations and surgeons, and control groups consisting of traditional implantation in order to truly determine the effects of customized implantation on unicompartmental knee arthroplasty.

## Figures and Tables

**Table 1:** Patient demographics.

**Number of Knees Included in Revision Rate Analysis**	*n* = 349 (287 medial, 62 lateral)	
**Number of knees available for follow-up and outcome analysis**	*n* = 242	
**Average time to follow-up**	4.2 years (range of 0.1–8.7)	
**Gender**	40% female	60% male
**Age at surgery**	71.1 years (SD: 9.2)	
**Body mass index (BMI)**	28.8 kg/m^2^ (SD: 4.7)	

## Data Availability

Data sharing is not applicable to this article. All data and findings have been presented in the above paper.
